# Supplementary material for: Entropy and crystal-facet modulation of P2-type layered cathodes for long-lasting sodium-based batteries
Source: Nat Commun. 2022 May 20;13:2826. doi: 10.1038/s41467-022-30113-0 (PMC9123165; doi:10.1038/s41467-022-30113-0)
Supplement: Supplementary file 1 — Supplementary Information [file 41467_2022_30113_MOESM1_ESM.pdf]

Supplementary Information for

Entropy and crystal-facet modulation of P2-type layered cathodes for long-lasting sodium-based batteries

Fang Fu,[1,6]* Xiang Liu,[2,6] Xiaoguang Fu,[1,6] Hongwei Chen,[1] Ling Huang,[3] Jingjing Fan,[3] Jiabo Le,[3] Qiuxiang Wang,[1] Weihua Yang,[1] Yang Ren,[4] Khalil Amine,[2,5]* Shi-Gang Sun[3]* and Gui-Liang Xu[2]*

[1] College of Materials Science and Engineering, Huaqiao University, Xiamen 361021, People's Republic of China

[2] Chemical Sciences and Engineering Division, Argonne National Laboratory, Lemont, IL 60439, USA

[3] Department of Chemistry, College of Chemistry and Chemical Engineering, Xiamen University, Xiamen 361005, China

[4] X-ray Science Division, Advanced Photon Source, Argonne National Laboratory, Lemont, IL 60439, USA

[5] Materials Science and Engineering, Stanford University, Stanford, CA, USA

[6] Fang Fu, Xiang Liu and Xiaoguang Fu contributed to this work equally

Supplementary Figures

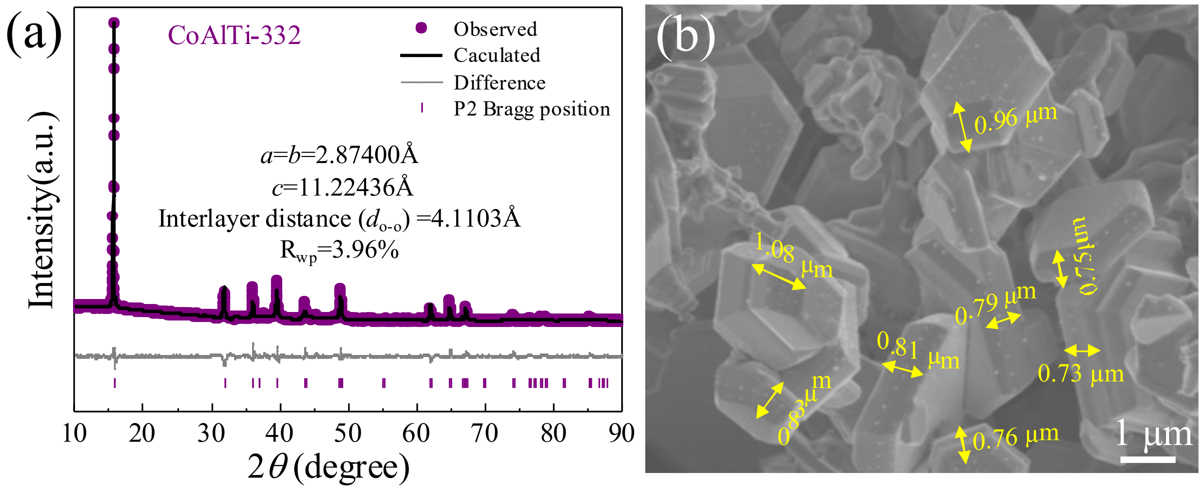

Supplementary Figure 1| (a) XRD pattern, Rietveld refinement plot and (b) SEM image of $Na_{0.62}Mn_{0.67}Ni_{0.23}Co_{0.033}Al_{0.033}Ti_{0.02}O_2$ (CoAlTi-332).

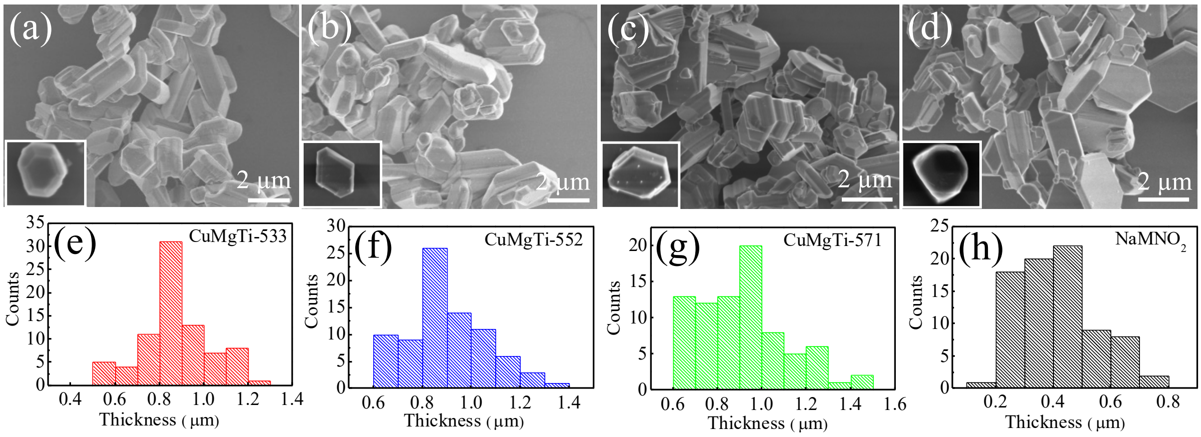

Supplementary Figure 2| SEM images and particle-thickness distribution of the (a, e) CuMgTi-533, (b, f) CuMgTi-552, (c, g) CuMgTi-571 and (d, h) $NaMNO_2$ samples.

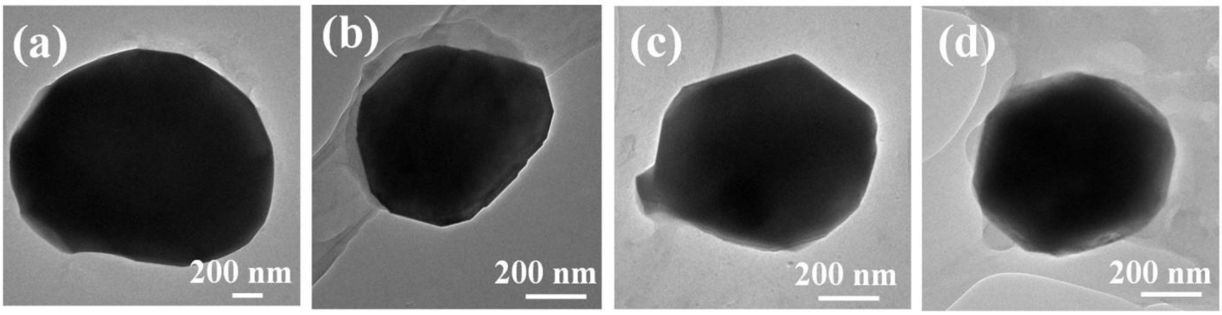

Supplementary Figure 3| TEM images of the (a) CuMgTi-533, (b) CuMgTi-552, (c) CuMgTi-571 and (d) $NaMNO_2$ particles.

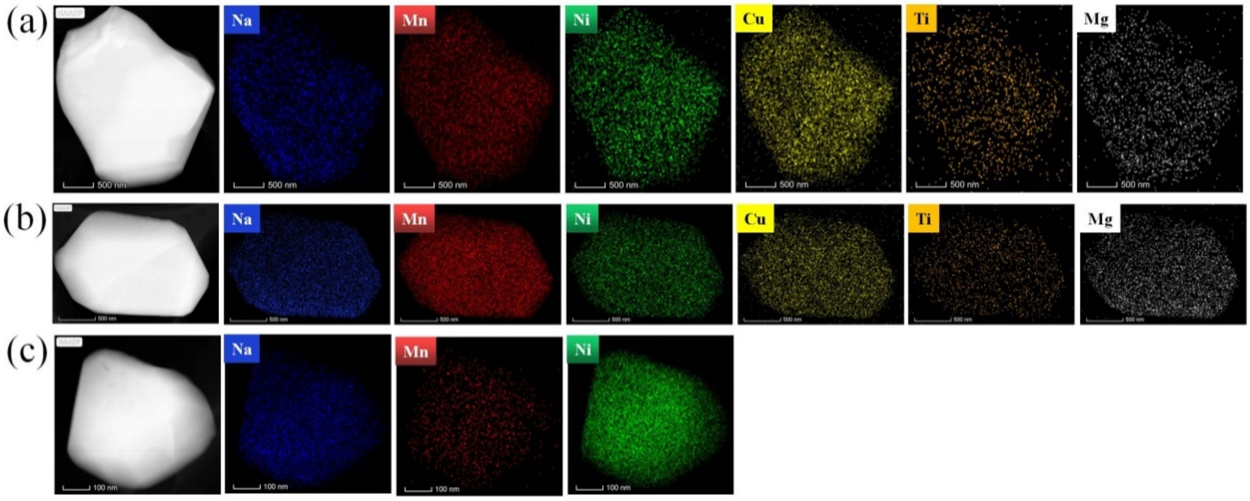

Supplementary Figure 4| HAADF-STEM images and the corresponding EDX elemental mappings of Na, Mn, Ni, Cu, Ti and Mg in the (a) CuMgTi-533, (b) CuMgTi-552, and (d) $NaMNO_2$ particles.

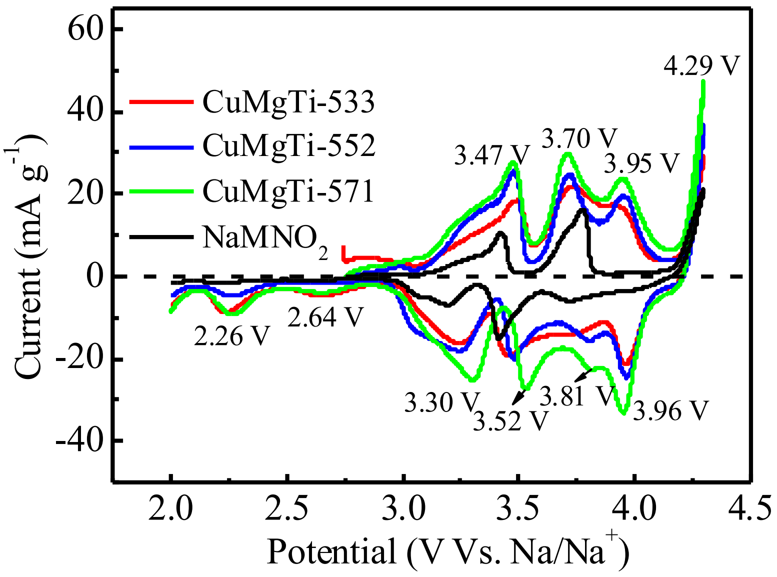

Supplementary Figure 5| CV curves of $Na_{0.62}Mn_{0.67}Ni_{0.23}Cu_{0.05}Mg_{0.09-2y}Ti_yO_2$ and $NaMNO_2$ electrodes between 2.0 and 4.3 V at 0.2 mV s^{-1} scan rate.

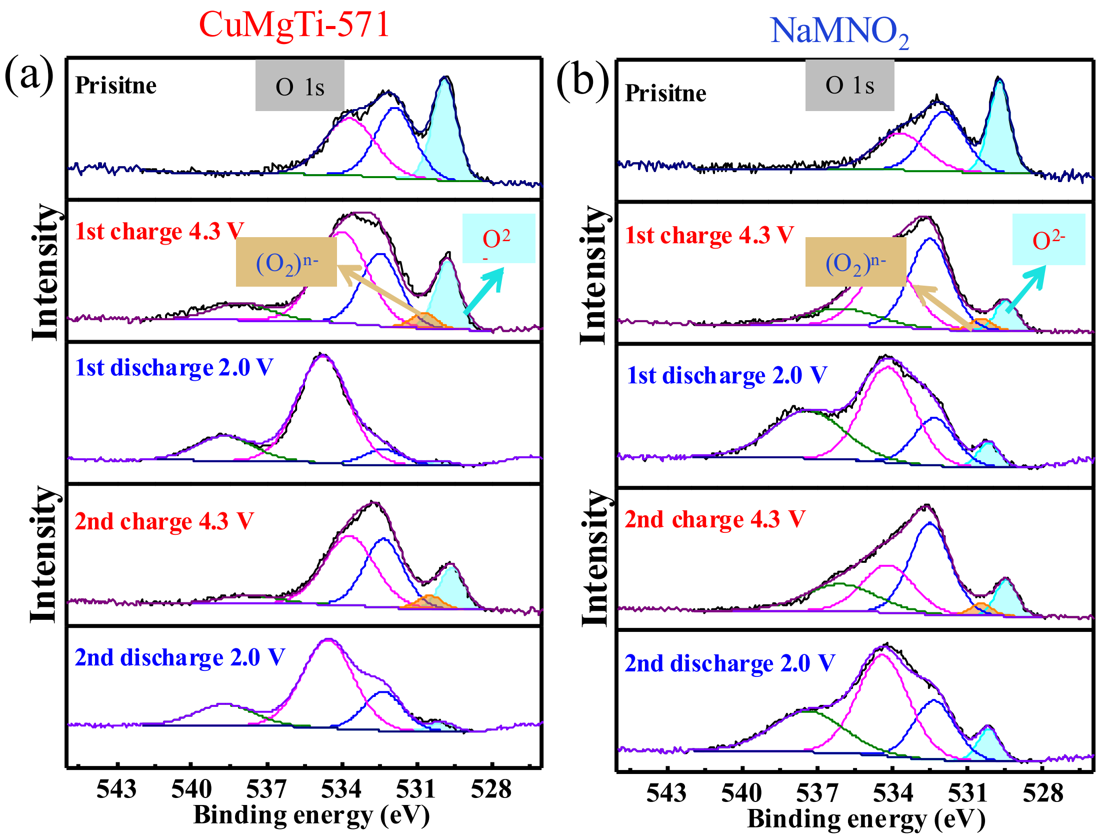

Supplementary Figure 6| O 1*s* XPS spectra collected for the CuMgTi-571 and $NaMNO_2$ samples at different charge-discharge states during the first two cycles.

Tarascon and co-workers have proved that XPS is an effective tool to probe the oxygen-related species.[1-3] We carried out O1*s* XPS experiments on CuMgTi-571 and $NaMNO_2$ samples to investigate the possible participation of lattice O^{2-} in the electrochemical process. Supplementary Figure 6 displays the XPS results of two samples charged and discharged at different voltages during the first two cycles. In the pristine samples, the peak located at ~529.5 eV can be attributed to the lattice O^{2-} while the additional peaks mainly originate from the weakly adsorbed oxygen surface species.[1-3] After charging to 4.3 V, a new component appears at 530.5 eV, which is ascribed to the oxidation process of $O^{2-}\rightarrow(O_2)^{n-}$ ($1\leq n\leq 3$).[3-5] This component disappears after discharged to 2.0 V, indicating the reversible reduction of $(O_2)^{n-}$. The component at 530.5 eV reappears/disappears upon second charges/discharge, further demonstrating the occurrence of anionic redox in CuMgTi-571 and $NaMNO_2$.

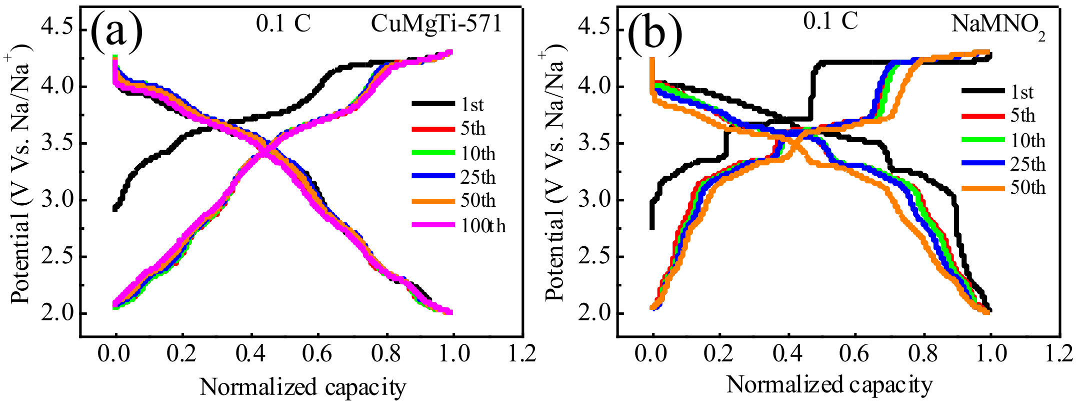

Supplementary Figure 7| Normalized charge/discharge profiles of (a) CuMgTi-571 and (b) $NaMNO_2$ at 0.1 C (1C= 120 mA g^{-1}) rate.

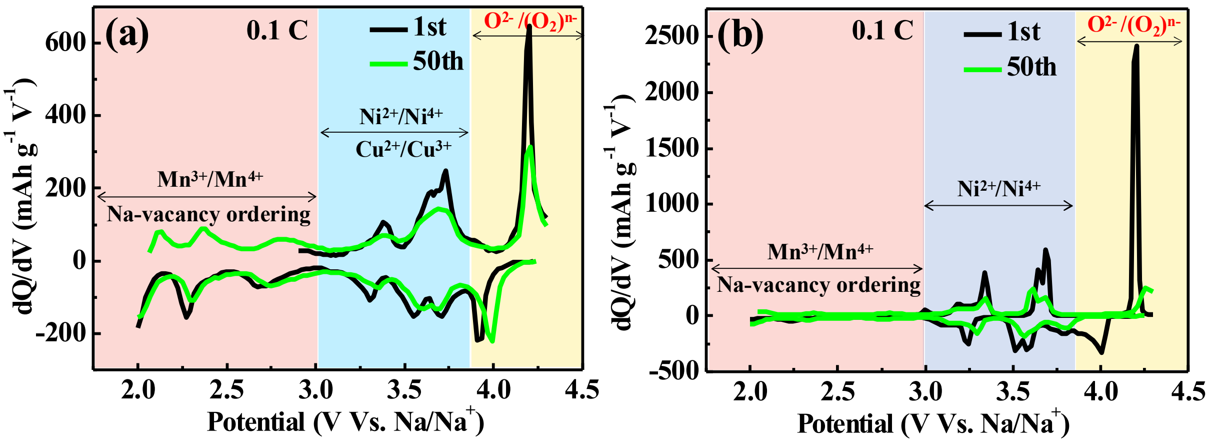

Supplementary Figure 8| Differential capacity vs. voltage (dQ/dV) plots of (a) CuMgTi-571 and (b) $NaMNO_2$ in the 1st and 50th cycle at 0.1 C (1C= 120 mA g^{-1}) rate.

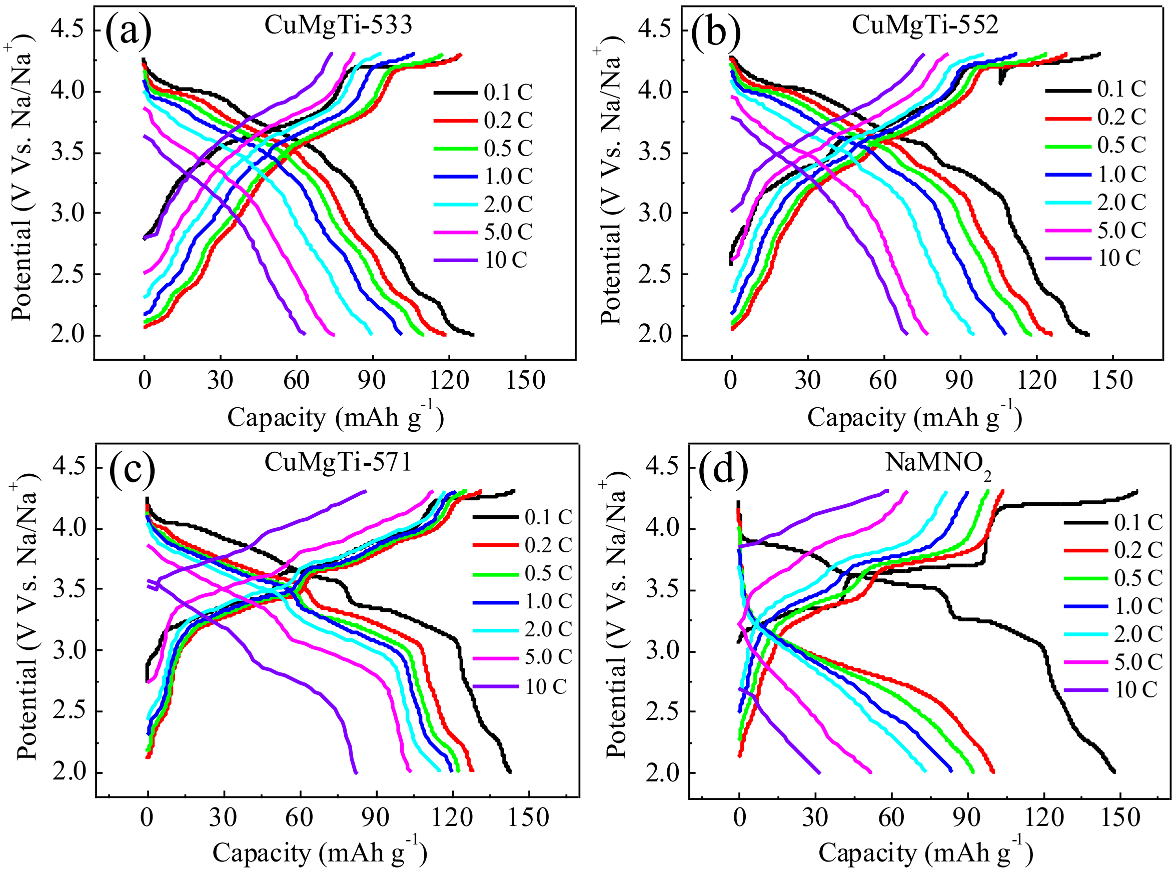

Supplementary Figure 9| Charge/discharge profiles of $Na_{0.62}Mn_{0.67}Ni_{0.23}Cu_{0.05}Mg_{0.09-2y}Ti_yO_2$ and $NaMNO_2$ at different C-rates within 2.0-4.3 V (1C= 120 mA g^{-1}).

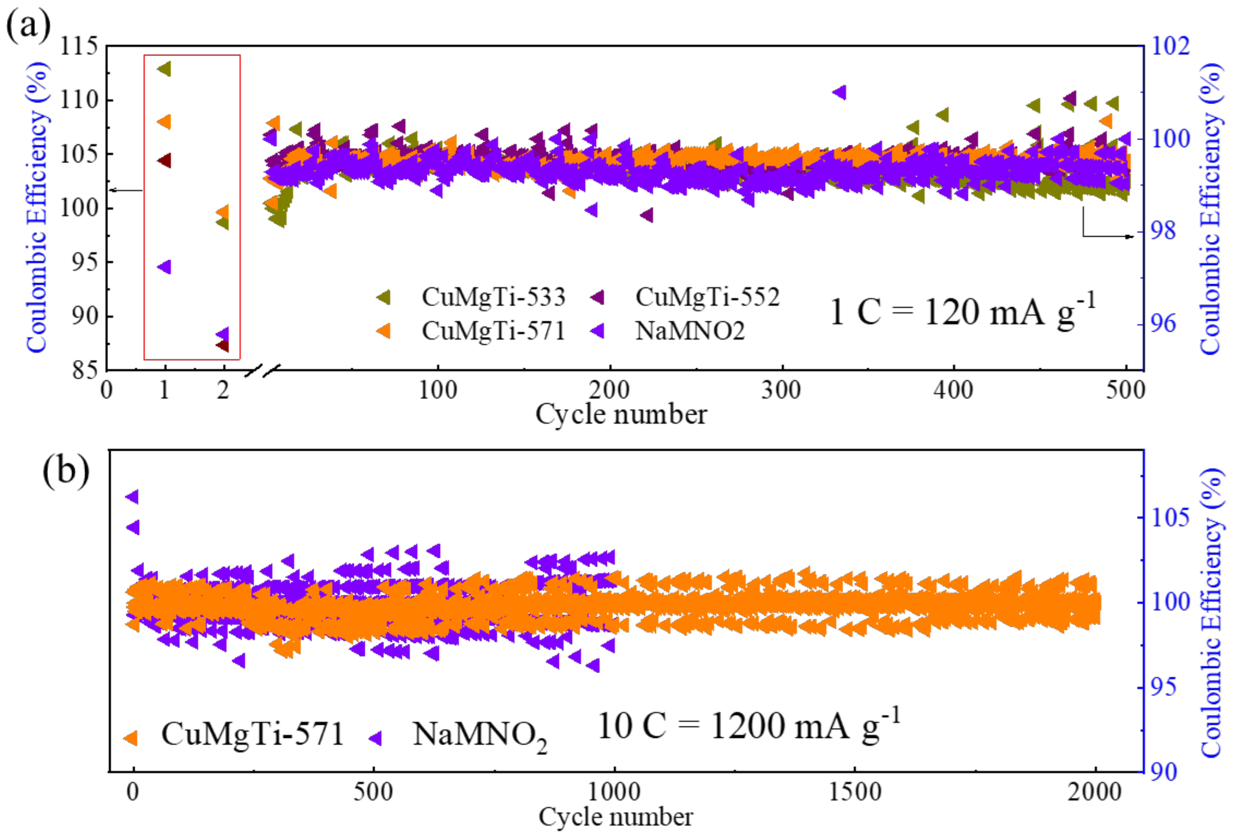

Supplementary Figure 10| (a) The Coulombic efficiency of $Na_{0.62}Mn_{0.67}Ni_{0.23}Cu_{0.05}Mg_{0.09-2y}Ti_yO_2$ and $NaMNO_2$ cathode during cycling at 1 C (120 mA g^{-1}). Left y-axis shows the Coulombic efficiency for the first two cycles, while the right y-axis shows the coulombic efficiency of the 3rd-500th cycle. (b) The Coulombic efficiency of CuMgTi-571 and $NaMNO_2$ cathode during cycling at 10 C (1200 mA g^{-1}).

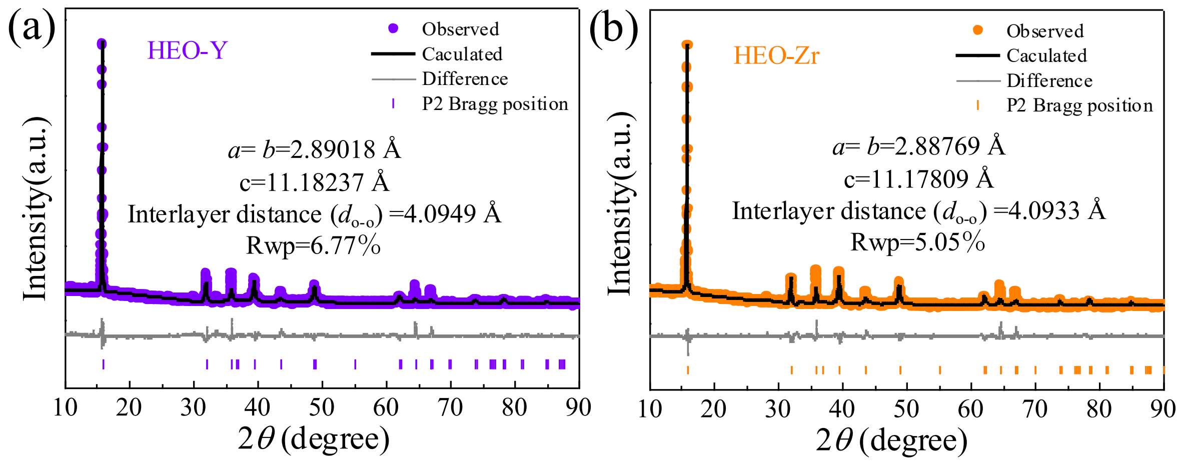

Supplementary Figure 11| The XRD patterns of (a) $Na_{0.62}Mn_{0.67}Ni_{0.21}Cu_{0.05}Mg_{0.016}Zn_{0.016}Sn_{0.016}Y_{0.016}O_2$ and (b) $Na_{0.62}Mn_{0.67}Ni_{0.21}Cu_{0.05}Mg_{0.015}Zn_{0.015}Sn_{0.015}Zr_{0.015}O_2$ samples.

The XRD patterns of $Na_{0.62}Mn_{0.67}Ni_{0.21}Cu_{0.05}Mg_{0.016}Zn_{0.016}Sn_{0.016}Y_{0.016}O_2$ and $Na_{0.62}Mn_{0.67}Ni_{0.21}Cu_{0.05}Mg_{0.015}Zn_{0.015}Sn_{0.015}Zr_{0.015}O_2$ samples are shown in Supplementary Figure 11. All diffraction peaks in the patterns can be indexed to the P2-type hexagonal structure without any impurity or secondary phase. The pure P2-type structures of the two samples suggest that eight metal ions with different radius are successfully incorporated into a single lattice. The interlayer distances (d_{o-o}) for HEO-Y and HEO-Zr obtained from the refinement are 4.0949 Å and 4.0933 Å, respectively. The d_{o-o} of HEO-Y is close to that of HEO-Zr, although the radius of Y^{3+} (0.9 Å) is much larger than that of Zr^{4+} (0.72).

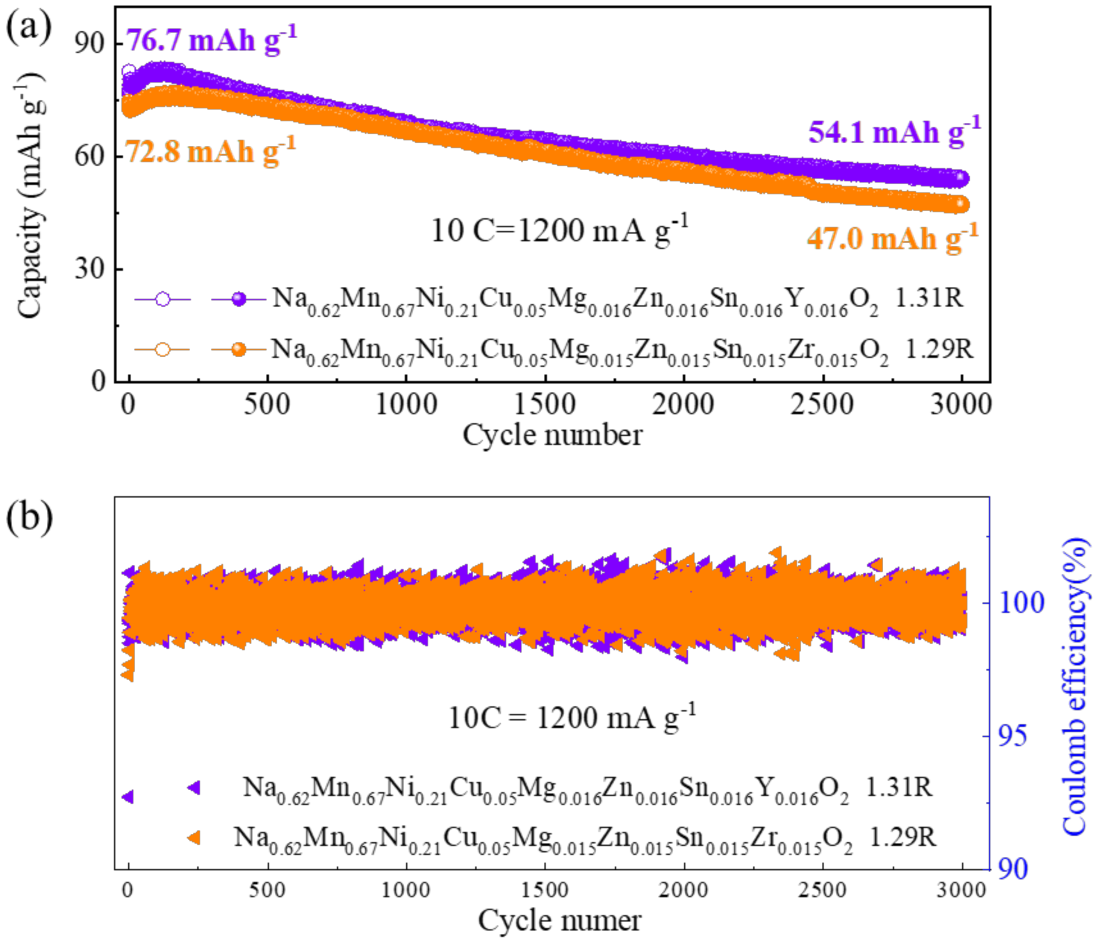

Supplementary Figure 12| (a) Cycling performance and (b) Coulombic efficiency of $Na_{0.62}Mn_{0.67}Ni_{0.21}Cu_{0.05}Mg_{0.016}Zn_{0.016}Sn_{0.016}Y_{0.016}O_2$ and $Na_{0.62}Mn_{0.67}Ni_{0.21}Cu_{0.05}Mg_{0.015}Zn_{0.015}Sn_{0.015}Zr_{0.015}O_2$ cathodes in coin cell configuration within 2.0-4.3 V versus Na/Na^+ at 10 C (1200 mA g^{-1}) and 25 °C. Hollow and solid circles in (a) represent charge and discharge capacity, respectively. Triangles in (b) represent coulombic efficiency.

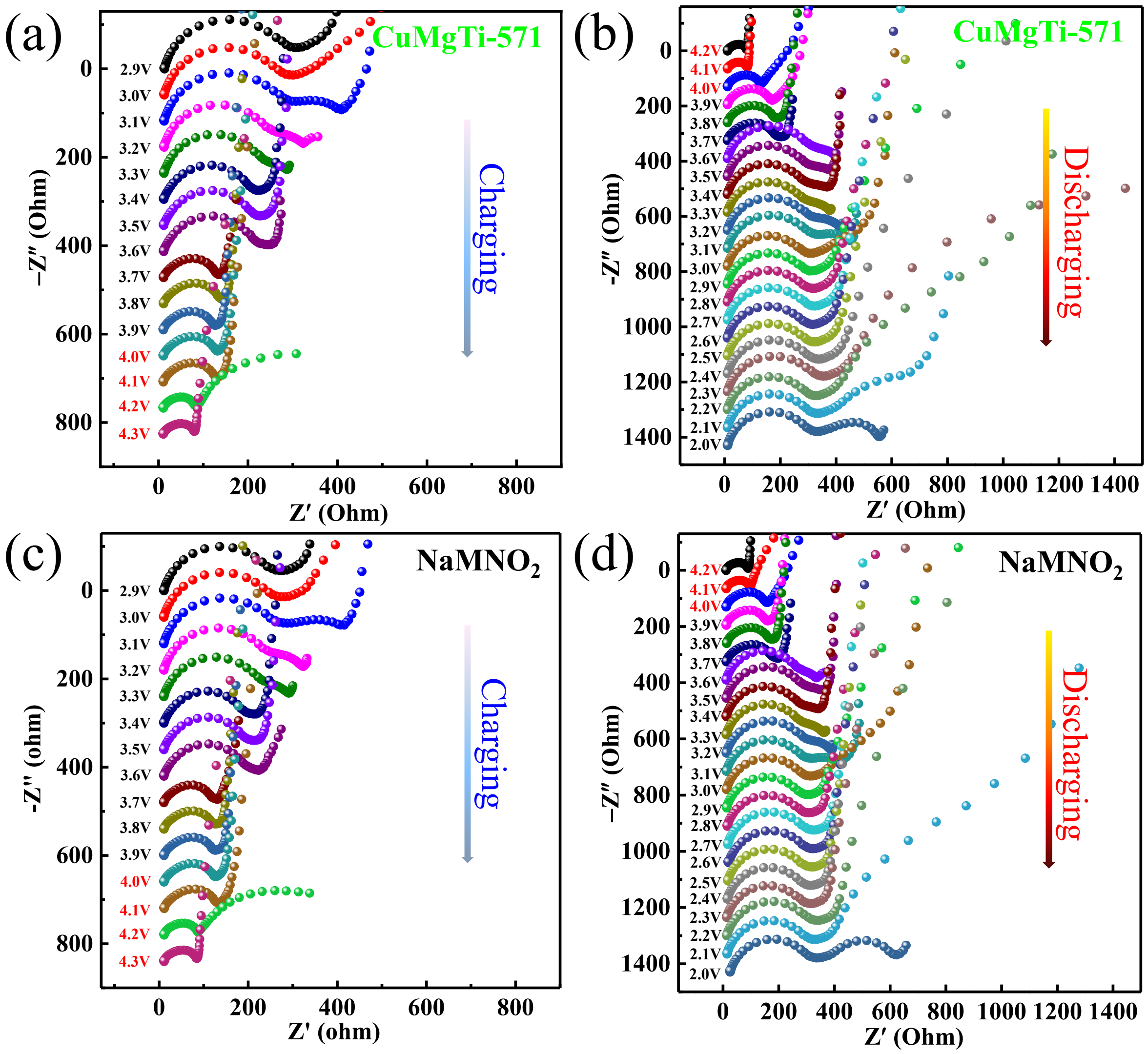

Supplementary Figure 13| Electrochemical impedance spectroscopy measurements of (a, b) CuMgTi-571 and (c, d) $NaMNO_2$ during the first charge and discharge process in coin cell with Na anode at 25 °C.

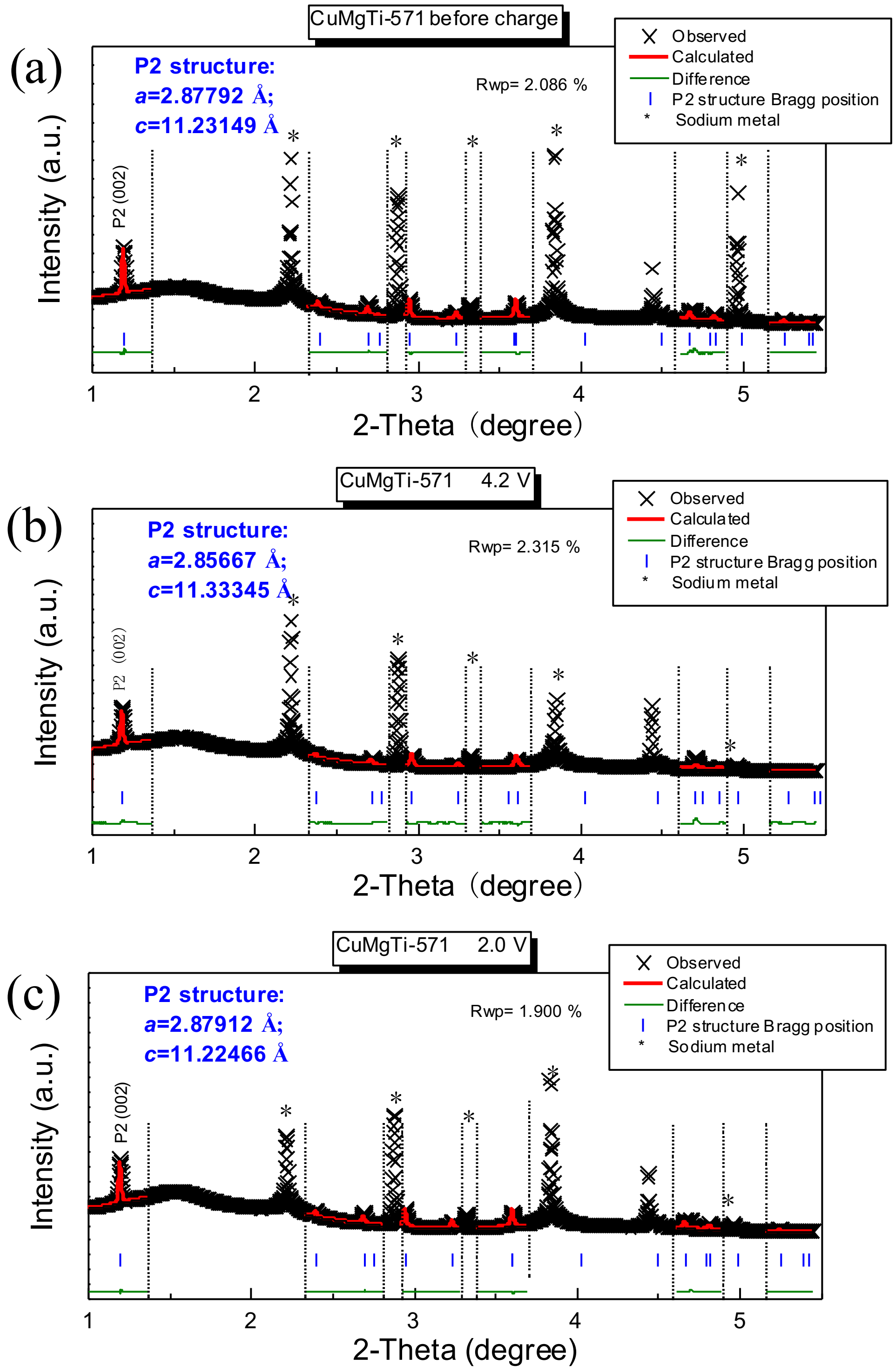

Supplementary Figure 14| The Rietveld refinement patterns of the in situ XRD data for CuMgTi-571 at various charged/discharged states: (a) before charge, (b) after charged to 4.2 V, (b) after discharged to 2.0 V.

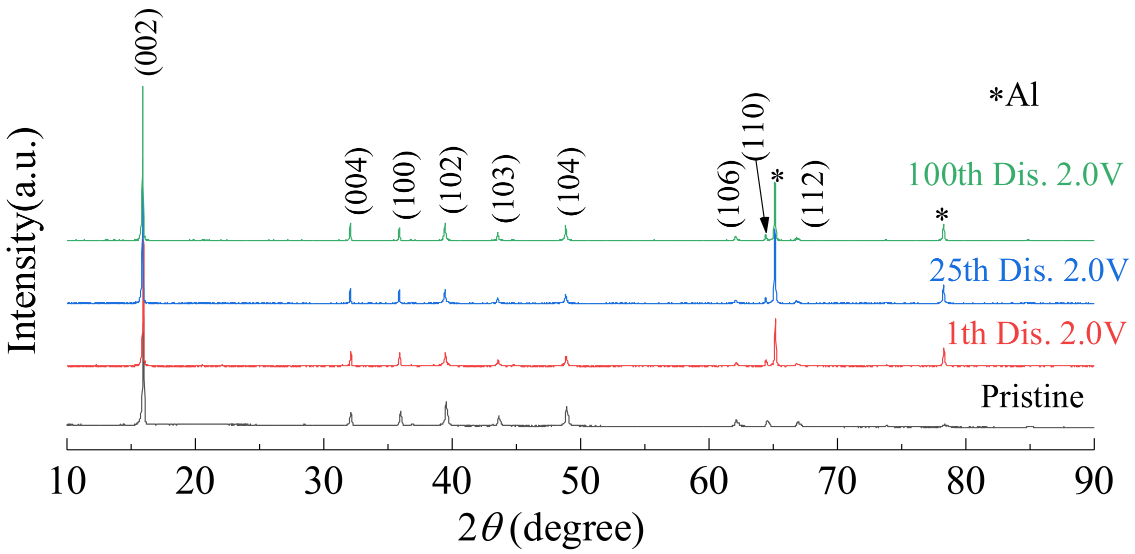

Supplementary Figure 15| XRD patterns of CuMgTi-571 tested at different cycles at 1.0 C (120 mA g^{-1}). Dis. represents discharge process.

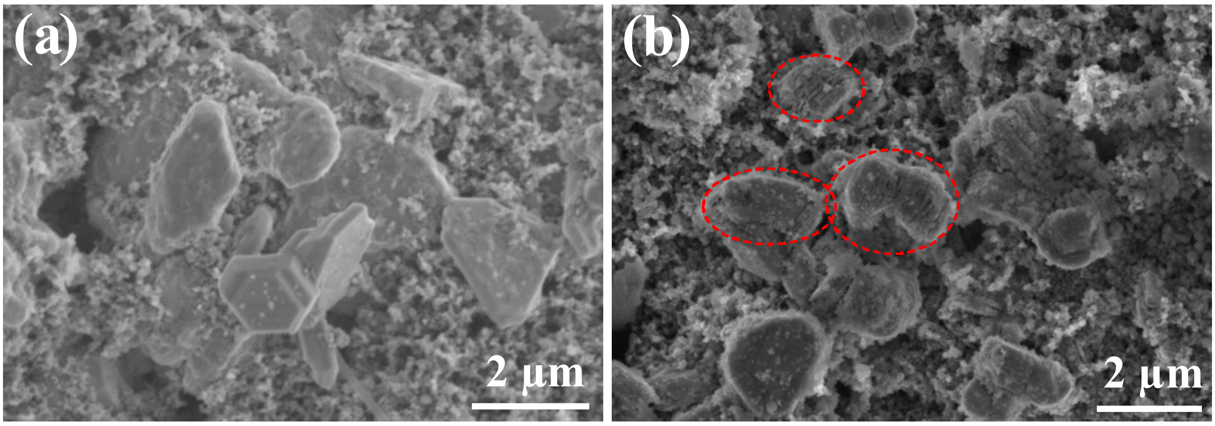

Supplementary Figure 16| Low magnification SEM images of the (a) CuMgTi-571 and (b) $NaMNO_2$ cathodes in the discharged state of 2.0 V after 500 cycles at 1.0 C (120 mA g^{-1}). The cycling tests were conducted in coin cell with Na anode between 2.0-4.3 V at 25 °C.

Supplementary Tables

Supplementary Table 1. Summary of diffusion coefficients of the Na-based layered oxides and Li-based layered oxides reported in the literatures using GITT tests. [6-18]

Compound	*D* (cm^2 s^{-1})	Ref.
O3-$LiNi_{0.8}Co_{0.1}Mn_{0.1}O_2$@PANI–PVP	0.5×10^{-8}-3.5×10^{-8}	6
O3-$LiNi_{0.88}Co_{0.09}Al_{0.03}O_2$-Te 1%	10^{-9}	7
O3-$LiNi_{0.6}Co_{0.2}Mn_{0.2}O_2$	10^{-9}	8
O3-$LiNi_{0.9}Co_{0.07}Al_{0.03}O_2$	10^{-9}	9
O3-$Li_{1.2}Mn_{0.54}Ni_{0.13}Co_{0.13}O_{2+\delta-x}F_x$	2×10^{-10}-8×10^{-10}	10
O3-$LiNi_{0.90}Co_{0.07}Mg_{0.03}O_2$	4×10^{-10}	11
O3-$Li_{1.5}[Mn_{0.75}Ni_{0.15}Co_{0.10}]O_{2+\delta}$	10^{-10}	12
P2-$Na_{2/3}Ni_{0.25}Mg_{0.083}Mn_{0.55}Ti_{0.117}O_2$	0.3×10^{-11}-0.75×10^{-11}	13
P2–$Na_{2/3}Ni_{1/3}Mn_{5/9}Al_{1/9}O_2$/RGO	1.54×10^{-11}	14
P2–$Na_{0.65}Li_{0.08}Cu_{0.08}Fe_{0.24}Mn_{0.6}O_2$	10^{-12}	15
P2–$Na_{0.66}Ni_{0.26}Zn_{0.07}Mn_{0.67}O_2$/0.06ZnO	10^{-12}	16
P2–$Na_xFe_{1/2}Mn_{1/2}O_2$	2.0×10^{-13}	17
P2–$Na_{0.6}Mn_{0.7}Ni_{0.3}O_{1.95}F_{0.05}$	10^{-14}	18

To investigate and compare the Li^+ kinetics and Na^+ kinetics in electrode materials, the diffusion coefficients of the common P2 Na-based layered oxides and Li-based layered oxides studied in the literatures are summarized and listed in Supplementary Table 1. It can be seen that the Na^+ diffusion coefficients in many P2 Na-based layered oxides are generally lower than 10^{-10} cm^2 s^{-1}, which are smaller than those of the Li-based layered oxides (mostly at magnitudes of $\approx10^{-8}$–10^{-10} cm^2 s^{-1}). Thus, Na^+ diffusion dynamics is in general slower than Li^+ diffusion dynamics in P2 layered cathode materials, owing to the larger size of Na^+.

Supplementary Table 2. Crystallographic details of the CuMgTi-533 obtained from Rietveld analysis.

CuMgTi-533					
Space group = $P6_3/mmc$					
$a = b = 2.8854 \pm 0.0002$ Å, $c = 11.1884 \pm 0.0009$ Å, $V = 93.15 \pm 0.01$ Å^3, R_{wp}=9.00					
atom	site	x	y	z	occupancy
Na	2d	1/3	2/3	3/4	0.4
Na	2b	0	0	1/4	0.22
Mn	2a	0	0	0	0.65
Ni	2a	0	0	0	0.23
Cu	2a	0	0	0	0.05
Mg	2a	0	0	0	0.03
Ti	2a	0	0	0	0.02
O	4f	1/3	2/3	0.06710	1

Supplementary Table 3. Crystallographic details of the CuMgTi-552 obtained from Rietveld analysis.

CuMgTi-552					
Space group = $P6_3/mmc$					
$a = b$ = 2.8881 ± 0.0001 Å, c = 11.1822 ± 0.0006 Å, V = 93.277 ± 0.006 Å^3, R_{wp}=8.707					
atom	site	x	y	z	occupancy
Na	2d	1/3	2/3	3/4	0.4
Na	2b	0	0	1/4	0.22
Mn	2a	0	0	0	0.69
Ni	2a	0	0	0	0.25
Cu	2a	0	0	0	0.05
Mg	2a	0	0	0	0.05
Ti	2a	0	0	0	0.02
O	4f	1/3	2/3	0.06988	1

Supplementary Table 4. Crystallographic details of the CuMgTi-571 obtained from Rietveld analysis.

CuMgTi-571					
Space group = $P6_3/mmc$					
$a = b = 2.8886 \pm 0.0002$ Å, $c = 11.1819 \pm 0.0007$ Å, $V = 93.303 \pm 0.008$ Å^3, R_{wp}=9.805					
atom	site	x	y	z	occupancy
Na	2d	1/3	2/3	3/4	0.4
Na	2b	0	0	1/4	0.22
Mn	2a	0	0	0	0.67
Ni	2a	0	0	0	0.24
Cu	2a	0	0	0	0.05
Mg	2a	0	0	0	0.07
Ti	2a	0	0	0	0.01
O	4f	1/3	2/3	0.06690	1

Supplementary Table 5. Crystallographic details of the $NaMNO_2$ obtained from Rietveld analysis.

$NaMNO_2$					
Space group = $P6_3/mmc$					
$a = b = 2.8824 \pm 0.0002$ Å, $c = 11.168 \pm 0.001$ Å, $V = 92.79 \pm 0.013$ Å^3, R_{wp}=9.96					
atom	site	*x*	*y*	*z*	occupancy
Na	2d	1/3	2/3	3/4	0.4
Na	2b	0	0	1/4	0.22
Mn	2a	0	0	0	0.66
Ni	2a	0	0	0	0.35
O	4f	1/3	2/3	0.07021	1

Supplementary Table 6. ICP results of $Na_{0.62}Mn_{0.67}Ni_{0.23}Cu_{0.05}Mg_{0.09-2y}Ti_yO_2$ and $NaMNO_2$ samples.

**Samples**	**Na**	**Mn**	**Ni**	**Cu**	**Mg**	**Ti**
CuMgTi-533	0.62	0.65	0.23	0.05	0.03	0.02
CuMgTi-552	0.62	0.69	0.25	0.05	0.05	0.02
CuMgTi-571	0.62	0.67	0.24	0.05	0.07	0.01
$NaMNO_2$	0.62	0.66	0.35			

Supplementary Table 7. Comparison of electrochemical performance of this work with other reported P2 layered cathodes tested at high rate.[19-24] S_{config} represents configurational entropy.

Cathode material	Entropy (S_{config})	Voltage range	Specific current	Cycle number	Capacity retention	Rate property (discharge capacity/ specific current)	Ref.
$Na_{0.62}Mn_{0.67}Ni_{0.23}Cu_{0.05}Mg_{0.07}Ti_{0.01}O_2$	1.28R	2.0-4.3 V	1200 mA g^{-1} (10 C)	2000<br>1000<br>500	75.4%<br>87.4%<br>95.4%	78.6 mAh g^{-1} /1200 mA g^{-1}	This work
$Na_{45/54}Li_{4/54}Ni_{16/54}Mn_{34/54}O_2$	1.00R	2.0-4.0 V	480 mA g^{-1}	3000	68%	60 mAh g^{-1} /1600 mA g^{-1}	19
$Na_{0.7}Mn_{0.6}Ni_{0.2}Mg_{0.2}O_2$	1.20R	2.5-4.2 V	170 mA g^{-1}	1000	79%	70 mAh g^{-1} /1700 mA g^{-1}	20
$Na_{2/3}Ni_{1/6}Mn_{2/3}Cu_{1/9}Mg_{1/18}O_2$	1.24R	2.5-4.15 V	600 mA g^{-1}	500	81.4%	78 mAh g^{-1} /1200 mA g^{-1}	21
$Na_{0.76}Cu_{0.22}Fe_{0.30}Mn_{0.48}O_2$	1.26R	2.0-4.0 V	240 mA g^{-1}	300	79%	64.9 mAh g^{-1} /1200 mA g^{-1}	22
$Na_{2/3}Mn_{0.72}Cu_{0.22}Mg_{0.06}O_2$	1.01R	2.0-4.5 V	174 mA g^{-1}	100	87.9%	70.3 mAh g^{-1} /1740 mA g^{-1}	23
$Na_{0.67}Mn_{0.71}Cu_{0.02}Mg_{0.02}Ni_{0.25}O_2$	1.01R	1.5-4.5 V	200 mA g^{-1}	100	81.9%	72 mAh g^{-1} /2000 mA g^{-1}	24

Supplementary Table 8. Comparison of the cycling performance of various sodium-based P2 layered cathodes with CuMgTi-571 tested at same cut-off voltage.[25-33] NA represents no available.

Cell configuration	Voltage range	Testing Tempe-rature	Specific current	Cycling performance	Ref.
Na| 1M $NaClO_4$ (in PC:FEC; 49:1 in volume) | $Na_{0.62}Mn_{0.67}Ni_{0.23}Cu_{0.05}Mg_{0.07}Ti_{0.01}O_2$	2.0-4.3 V	25 °C	12 mA g^{-1} (0.1 C)	89.6% (132.9 mAh g^{-1}) after 100 cycles	This work
Na| 1M $NaClO_4$ (in PC:FEC; 49:1 in volume) | $Na_{0.62}Mn_{0.67}Ni_{0.23}Cu_{0.05}Mg_{0.07}Ti_{0.01}O_2$	2.0-4.3 V	25 °C	120 mA g^{-1} (1 C)	87% (103.3 mAh g^{-1}) after 500 cycles	This work
Na| 1M $NaClO_4$ (in PC:EC; 1:1 in volume) | $Na_{2/3}Ni_{1/3}Mn_{2/3}O_2$	2.0-4.3 V	25 °C	170 mA g^{-1}	59.93% (53.9 mAh g^{-1}) after 100cycles	25
Na| 1M $NaClO_4$ (in EC:DEC; 1:1 in volume) | $Na_{0.67}Ni_{0.17}Ti_{0.16}Mn_{0.67}O_2$	1.5-4.3 V	NA	1.0 C	67% (113.9 mAh g^{-1}) after 100cycles.	26
Na| 0.5M $NaPF_6$ (in PC:FEC; 97:3 in volume) | Rhenanite-coated $Na_{2/3}[Ni_{1/3}Mn_{2/3}]O_2$	2.5-4.3 V	25 °C	40 mA g^{-1}	74% (111 mAh g^{-1}) after 200 cycles	27
Na| 0.5M $NaPF_6$ (in PC:FEC; 98:2 in volume) | $Na_{0.55}[Ni_{0.1}Fe_{0.1}Mn_{0.8}]O_2$	1.5-4.3 V	30 °C	60 mA g^{-1}	75% (102.9 mAh g^{-1}) after 100 cycles	28
Na| 1M $NaClO_4$ (in EC:DMC; 1:1 involume) | $Na_{0.67}[Mn_{0.67}Ni_{0.21}Li_{0.06}Zn_{0.06}]O_2$	2.0-4.3 V	NA	173 mA g^{-1}	75% (137.3 mAh g^{-1}) after 500 cycles	29
Na| 1M $NaClO_4$ (in PC:FEC; 95:5 in volume) | Co gradient $Na_{2/3}[Ni_{1/3}Mn_{2/3}]O_2$	2.0-4.3 V	25 °C	16 mA g^{-1}	77.4% (127.7 mAh g^{-1}) after 100cycles	30
Na| 1M $NaClO_4$ (in PC:FEC; 95:5 in volume) | $Na_{0.67}Ni_{0.33}Mn_{0.47}Ti_{0.2}O_2$	2.5-4.3 V	NA	173 mA g^{-1}	78.6% (88.1 mAh g^{-1}) after 200 cycles	31
Na| 1M $NaClO_4$ (in PC:FEC; 95:5 in volume) | $Na_{2/3}[Ni_{0.3}Co_{0.1}Mn_{0.6}]O_2$	2.0-4.3 V	25 °C	15 mA g^{-1}	79.2% (127.9 mAh g^{-1}) after 50 cycles	32
Na| 1M $NaClO_4$ (in EC:DEC; 1:1 in volume) | $Na_{0.66}Co_{0.22}Mn_{0.44}Ti_{0.34}O_2$	1.5-4.3 V	25 °C	35.4 mA g^{-1}	79.4% (104 mAh g^{-1}) after 100 cycles	33

Supplementary Table 9. Diameters of semicircles in EIS spectra of CuMgTi-571 and $NaMNO_2$ at high voltage region (4.0-4.3 V). CC and DC represent the charge and discharge process, respectively.

High-voltage region	Diameter of semicircle in EIS spectra of CuMgTi-571	Diameter of semicircle in EIS spectra of $NaMNO_2$
CC 4.0 V	132.6 Ω	131.8 Ω
CC 4.1 V	133.8 Ω	138.0 Ω
CC 4.2 V	86.5 Ω	89.5 Ω
CC 4.3 V	78.7 Ω	84.2 Ω
DC 4.2 V	73.9 Ω	87 Ω
DC 4.1 V	75.7 Ω	98.6 Ω
DC 4.0 V	135.6 Ω	162 Ω

Supplementary Table 10. Comparison of volume change of this work with other reported P2 layered cathodes.[15,23,34-47]

Cathode material	Voltage range	Specific current	Volume change after charge/discharge	Ref.
$Na_{0.62}Mn_{0.67}Ni_{0.23}Cu_{0.05}Mg_{0.07}Ti_{0.01}O_2$	2.0-4.3 V	24 mA g^{-1}	0.57% after charged to 4.3 V, 0.022% after first cycle	This work
$Na_{2/3}[Mn_{7/9}Mg_{1/9}\square_{1/9}]O_2$	1.5-4.5 V	15 mA g^{-1}	3.3% after first cycle	34
$Na_{2/3}MnO_2$	1.5-4.4 V	NA	2.5% after first cycle	35
$Na_xCo_{0.1}Mn_{0.9}O_{2+z}$	1.5-3.8 V	NA	2.3% after first cycle	36
$Na_{0.7}Fe_{0.4}Mn_{0.4}Co_{0.2}O_2$	2.0-4.5 V	80 mA g^{-1}	-2.1% after first cycle	37
$Na_{2/3}Mn_{1/2}Ni_{1/6}Co_{1/3}O_2$	1.5-4.5 V	20 mA g^{-1}	1.9% after first cycle	38
$Na_{0.85}Li_{0.12}Ni_{0.22}Mn_{0.66}O_2$	2.0-4.3 V	22.4 mA g^{-1}	1.7% after first cycle	39
$Na_{0.649}Al_{0.096}Mn_{0.826}\square_{0.078}O_2$	1.5-4.5 V	24 mA g^{-1}	-1.4% after first cycle	40
$Na_{2/3}Zn_{1/4}Mn_{3/4}O_2$	1.5-4.5 V	20 mA g^{-1}	1.0% after first cycle	41
$Na_{0.72}[Li_{0.24}Mn_{0.76}]O_2$	1.5-4.5 V	20 mA g^{-1}	0.92% after first cycle	42
$Na_{0.7}Mg_{0.2}[Fe_{0.2}Mn_{0.6}\square_{0.2}]O_2$	1.5-4.5 V	15 mA g^{-1}	0.86% after first cycle	43
$Na_{0.65}Li_{0.08}Cu_{0.08}Fe_{0.24}Mn_{0.6}O_2$	2.5-4.2 V	10 mA g^{-1}	0.7% after first cycle	15
$Na_{0.67}Li_{0.21}Mn_{0.59}Ti_{0.2}O_2$	1.5-4.5 V	10 mA g^{-1}	0.7% after first cycle	44
$Na_{2/3}Mn_{0.72}Cu_{0.22}Mg_{0.06}O_2$	2.0-4.5 V	17.4 mA g^{-1}	-0.68% after first cycle	23
$Li_{0.1}Na_{0.57}Ni_{0.33}Mn_{0.67}O_2$	2.0-4.0 V	NA	-0.64% after first cycle	45
$Na_xK_{0.065}MnO_2$	1.8-4.3 V	NA	7.8% after two cycles	46
Na_xMnO_2	2.0-4.5 V	24 mA g^{-1}	1.96% after two cycles	47

Supplementary Notes

Supplementary Note 1: about configuration entropy

The structural stability and phase stability of materials rely on both the entropy (ΔS) and enthalpy (ΔH), according to the Gibbs free energy (ΔG) formula,[48] which is presented in Equation (1):

$$\Delta G = \Delta H - T\Delta S \tag{1}$$

Where ΔS is the total entropy change, including both configurational and dynamical contributions. The stable system always has the lowest Gibbs free energy. According to Equation (1), it can be inferred that increasing the configurational entropy (S_{config}) could result in large value of overall entropy and low Gibbs free energy, which is beneficial to improve the structural stability of materials. Thus, the structural stability can be mediated through manipulating a material's configurational entropy. This entropy-mediated structural stabilization could open up a new way for tailoring/optimizing the properties of electrode materials. The configurational entropy of the material can be calculated according to Equation (1) (in article), which can be used in ideal gas mixture, ideal or regular solution, and solid compound with single-phase crystal structure.[48-51] The contribution of the anion-site has a minor influence on S_{config}, thus, the effect of anion is not considered. This simple relationship between the configurational entropy and the number and mole fraction of elements serves as the basis for manipulating a material's configurational entropy. The configurational entropy can be manipulated by changing the number and mole fraction of constituent elements. Increasing the configurational entropy through incorporating multiple cations and tuning the molar fraction of constituent cations would be a powerful way to intrinsically stabilize the structure of electrode materials. However, until now the merits of entropy-mediated structural stabilization on mitigating and blocking the structural change and degradation of P2-structured layered cathode materials have not been observed.

Supplementary Note 2: Calculation method of Na^+ diffusion coefficient:

Based on the GITT measurement, the Na^+ diffusion coefficient can be calculated by Equation (2):

$$D_{ion} = \frac{4}{\pi\tau} \times \left(\frac{m_B V_M}{M_B S}\right) \times \left(\frac{\Delta E_S}{\Delta E_\tau}\right)^2 \tag{2}$$

where τ is the duration time of the current pulse (s), m_B is the weight of the active material (g), V_M is the molar volume of the unit cell (cm^3 mol^{-1}), M_B is the molecular weight of the sample (g mol^{-1}), S is the electrode/electrolyte contact area (cm^2), ΔE_s is the steady-state voltage change during the current pulse, and ΔE_τ is the variation of the cell voltage.[52,53]

Supplementary Note 3: Calculation method of molar volume of the unit cell (V_M):

Based on the Rietveld refined lattice parameters, the unit cell volume (V) of $Na_{0.62}Mn_{0.67}Ni_{0.23}Cu_{0.05}Mg_{0.09-2y}Ti_yO_2$ and $NaMNO_2$ can be calculated by "V=a×b×c× sin120°", thus 1 cm^3 has 1/V unit cells. Since each unit cell has two molecules, and each molecule has 0.62 Na^+, 1 cm^3 has $(1/V\times2\times0.62)/6.02\times10^{23}$ mol Na^+. Thus, Na^+ concentration in sample is $(1/V\times2\times0.62)/6.02\times10^{23}$ mol cm^{-3}, and V_M can be further deduced.[54]

References List

1. Sathiya, M. et al. Reversible anionic redox chemistry in high-capacity layered-oxide electrodes. *Nat. Mater.* **12,** 827-835 (2013).
2. Sathiya, M. et al. High performance $Li_2Ru_{1-y}Mn_yO_3$ ($0.2 \leq y \leq 0.8$) cathode materials for rechargeable lithium-ion batteries: their understanding. *Chem. Mater.* **25**, 1121-1131 (2013).
3. Bai, X. et al. Anionic redox activity in a newly Zn-doped sodium layered oxide P2-$Na_{2/3}Mn_{1-y}Zn_yO_2$ ($0 < y < 0.23$). *Adv. Energy Mater.* **8,** 1802379 (2018).
4. Cao, X. et al. Reversible oxygen redox chemistry in layered oxides for sodium-ion batteries. *Adv. Energy Mater.* **10**, 1903785 (2020).
5. Song, B. et al. Understanding the low-voltage hysteresis of anionic redox in $Na_2Mn_3O_7$. *Chem. Mater.* **31**, 3756-3765 (2019).
6. Gan, Q. et al. Polyvinylpyrrolidone-induced uniform surface-conductive polymer coating endows Ni-rich $LiNi_{0.8}Co_{0.1}Mn_{0.1}O_2$ with enhanced cyclability for lithium-ion batteries. *ACS Appl. Mater. Interfaces.* **11,** 12594-12604 (2019).
7. Huang, Y. et al. Tellurium surface doping to enhance the structural stability and electrochemical performance of layered Ni-rich cathodes. *ACS Appl. Mater. Interfaces.* **11,** 40022-40033 (2019).
8. Wang, Q. et al. Origin of structural evolution in capacity degradation for overcharged NMC622 via operando coupled investigation. *ACS Appl. Mater. Interfaces.* **9,** 24731-24742 (2017).
9. Zhou, P. et al. Stable layered Ni-rich $LiNi_{0.9}Co_{0.07}Al_{0.03}O_2$ microspheres assembled with nanoparticles as high-performance cathode materials for lithium-ion batteries. *J. Mater. Chem. A.* **5,** 2724-2731 (2017).
10. Cao, S. et al. Suppressing the voltage decay based on a distinct stacking sequence of oxygen atoms for Li-rich cathode materials. *ACS Appl. Mater. Interfaces.* **13,** 17639-17648 (2021).
11. Zhang, Y. et al. $LiNi_{0.90}Co_{0.07}Mg_{0.03}O_2$ cathode materials with Mg-concentration gradient for rechargeable lithium-ion batteries. *J. Mater. Chem. A.* **7,** 20958-20964 (2019).
12. Yu, R. et al. Self-assembly synthesis and electrochemical performance of $Li_{1.5}Mn_{0.75}Ni_{0.15}Co_{0.10}O_{2+\delta}$ microspheres with multilayer shells. *J. Mater. Chem. A.* **3,** 3120-3129 (2015).
13. Huang Y. et al. Vitalization of P2-$Na_{2/3}Ni_{1/3}Mn_{2/3}O_2$ at high-voltage cyclability via combined structural modulation for sodium-ion batteries. *Energy Storage Mater.* **29,** 182-189 (2020).
14. Zhang, X. et al. P2-$Na_{2/3}Ni_{1/3}Mn_{5/9}Al_{1/9}O_2$ microparticles as superior cathode material for sodium-ion batteries: enhanced properties and mechanism via graphene connection. *ACS Appl. Mater. Interfaces.* **8,** 20650-20659 (2016).
15. Qi, R. et al. A highly-stable layered Fe/Mn-based cathode with ultralow strain for advanced sodium-ion batteries. *Nano Energy.* **88,** 106206 (2021).
16. Zhang, F. et al. Stabilizing P2-type Ni-Mn oxides as high-voltage cathodes by a doping-integrated coating strategy based on zinc for sodium-ion batteries. *ACS Appl. Mater. Interfaces.* **13,** 40695-40704 (2021).
17. Li, M. et al. Eutectic synthesis of the P2-type $Na_xFe_{1/2}Mn_{1/2}O_2$ cathode with improved cell design for sodium-ion batteries. *ACS Appl. Mater. Interfaces.* **12,** 23951-23958 (2020).
18. Kang, W. et al. Tunable electrochemical activity of P2-$Na_{0.6}Mn_{0.7}Ni_{0.3}O_{2-x}F_x$ microspheres as high-rate cathodes for high-performance sodium ion batteries. *ACS Appl. Mater. Interfaces.* **13,** 15333-15343 (2021).

19. Zhao, C. et al. Revealing high Na-content P2-Type layered oxides as advanced sodium-ion cathodes. *J. Am. Chem. Soc.* **142,** 5742-5750 (2020).
20. Wang, Q. C. et al. Tuning P2-structured cathode material by Na-site Mg substitution for Na-ion batteries. *J. Am. Chem. Soc.* **141,** 840-848 (2019).
21. Xiao, Y. et al. A stable layered oxide cathode material for high-performance sodium-ion battery. *Adv. Energy Mater.* **9,** 1803978 (2019).
22. Shen, Q. et al. Dual-strategy of cation-doping and nanoengineering enables fast and stable sodium-ion storage in a novel Fe/Mn-based layered oxide cathode. *Adv. Sci.* **7,** 2002199 (2020).
23. Wang, P. et al. Both cationic and anionic redox chemistry in a P2-type sodium layered oxide. *Nano Energy.* **69**, 104474 (2020).
24. Kong, W. et al. Simultaneously tuning cationic and anionic redox in P2-$Na_{0.67}Mn_{0.75}Ni_{0.25}O_2$ cathode material through a synergic Cu/Mg co-doping. *J. Mater. Chem. A.* **7,** 9099-9109 (2019).
25. Zheng, X. et al. New insights into understanding the exceptional electrochemical performance of P2-Type manganese-based layered oxide cathode for sodium ion batteries. *Energy Storage Mater.* **15**, 257-265 (2018).
26. Pahari, D. et al. On controlling the P2-O2 phase transition by optimal Ti-substitution on Nisite in P2-type $Na_{0.67}Ni_{0.33}Mn_{0.67}O_2$ (NNMO) cathode for Na-ion batteries. *J. Power Sources.* **455,** 227957 (2020).
27. Jo, C. et al. Bioinspired surface layer for the cathode material of high-energy-density sodium-ion batteries. *Adv. Energy Mater.* **8,** 1702942 (2018).
28. Hwang, J. et al. A new P2-type layered oxide cathode with extremely high energy density for sodium-ion batteries. *Adv. Energy Mater.* **9,** 1803346 (2019).
29. Li, W. et al. Exploring the stability effect of the Co-substituted P2-$Na_{0.67}[Mn_{0.67}Ni_{0.33}]O_2$ cathode for liquid- and solid-state sodium-ion satteries. *ACS Appl. Mater. Interfaces.* **12,** 41477-41484 (2020).
30. Hou, P. et al. Mitigating the P2-O2 phase transition of high-voltage P2-$Na_{2/3}[Ni_{1/3}Mn_{2/3}]O_2$ cathode by cobalt gradient substitution for high-rate sodium-ion batteries. *J. Mater. Chem. A,* **7,** 4705-4713 (2019).
31. Tang, K. et al. Electrochemical performance and structural stability of air-stable $Na_{0.67}Ni_{0.33}Mn_{0.67-x}Ti_xO_2$ cathode materials for high-performance sodiumion batteries. *Chem. Eng. J.* **399,** 125725 (2020).
32. Hou, P. et al. A high energy-density P2-$Na_{2/3}[Ni_{0.3}Co_{0.1}Mn_{0.6}]O_2$ cathode with mitigated P2-O2 transition for sodium-ion batterie. *Nanoscale,* **11,** 2787-2794 (2019).
33. Wang, Q. et al. Utilizing Co^{2+}/Co^{3+} redox couple in P2-layered $Na_{0.66}Co_{0.22}Mn_{0.44}Ti_{0.34}O_2$ cathode for sodium-ion batteries. *Adv. Sci.* **4,** 1700219 (2017).
34. Yang, L. et al. Superiority of native vacancies in activating anionic redox in P2-type $Na_{2/3}[Mn_{7/9}Mg_{1/9}\square_{1/9}]O_2$. *Nano Energy*. **78,** 105172 (2020).
35. Kumakura, S. et al. Sodium and manganese stoichiometry of P2-Type $Na_{2/3}MnO_2$. *Angew. Chem. Int. Ed*. **55,** 12760 (2016).
36. Bucher, N. et al. P2-$Na_xCo_yMn_{1-y}O_2$ (y=0, 0.1) as cathode materials in sodium-ion batteries effects of doping and morphology to enhance cycling stability. *Chem. Mater.* **28**, 2041-2051 (2016).

37. Jung, Y. et al. In situ X-ray diffraction studies on structural changes of a P2 layered material during electrochemical desodiation/sodiation. *Adv. Funct. Mater*. **25,** 3227-3237 (2015).
38. Liu, Z. et al. Ultralow volume change of P2-type layered oxide cathode for Na-ion batteries with controlled phase transition by regulating distribution of Na^+. *Angew. Chem. Int. Ed*. **60,** 2-12 (2021).
39. Jin, T. et al. Realizing complete solid-solution reaction in high sodium content P2-type cathode for high-performance sodium-ion batteries. *Angew. Chem. Int. Ed*. **59,** 14511 (2020).
40. Xiao, Z. et al. Insights of the electrochemical reversibility of P2-type sodium manganese oxide cathodes via modulation of transition metal vacancies. *ACS Appl. Mater. Interfaces.* **13,** 38305-38314 (2021).
41. Wang, Y. et al. Ultralow-strain Zn-substituted layered oxide cathode with suppressed P2–O2 transition for stable sodium ion storage. *Adv. Funct. Mater.* **30,** 1910327 (2020).
42. Rong, X. et al. Anionic redox reaction-induced high-capacity and low-strain cathode with suppressed phase transition. *Joule* **3,** 503-517 (2019).
43. Li, X-. L. et al. Whole-voltage-range oxygen redox in P2-layered cathode materials for sodium-ion batteries. *Adv. Mater.* **33,** 2008194 (2021).
44. Xu, H. et al. Anion-cation synergetic contribution to high capacity, structurally stable cathode materials for sodium-ion batteries. *Adv. Funct. Mater.* **30,** 2005164 (2020).
34. Wang, P. et al. Both cationic and anionic redox chemistry in a P2-type sodium layered oxide. *Nano Energy* **69,** 104474 (2020).
45. Xie, Y. et al. Role of lithium doping in P2-$Na_{0.67}Ni_{0.33}Mn_{0.67}O_2$ for sodium-ion batteries. *Chem. Mater.* **33,** 4445-4455 (2021).
46. Wang, C. et al. Tuning local chemistry of P2 layered-oxide cathode for high energy and long cycles of sodium-ion battery. *Nat. Commun.* **12,** 2256 (2021).
47. Zuo, W. et al. Engineering Na^+-layer spacings to stabilize Mnbased layered cathodes for sodium-ion batteries. *Nat. Commun.* **12,** 4903 (2021).
48. Santodonato, L. et al. Deviation from high-entropy configurations in the atomic distributions of a multi-principal-element alloy. *Nat. Commun.* **6,** 5964 (2015).
49. Kushwaha, K. et al. A record chromophore density in high-entropy liquids of two low-melting perylenes: a new strategy for liquid chromophores. *Adv. Sci.* **6,** 1801650 (2019).
50. Abhishek Sarkar, A. et al. High-entropy oxides: fundamental aspects and electrochemical properties. *Adv. Mater.* **31,** 1806236 (2019).
51. Abhishek Sarkar, A. et al. High entropy oxides for reversible energy storage. *Nat. Commun.* **9,** 3400 (2018).
52. Su, B. et al. High-performance $NaVO_3$ with mixed cationic and anionic redox reactions for Na-ion battery applications. *Chem. Mater.* **32,** 8836-8844 (2020).
53. Zhang, H. et al. In situ synthesis of multilayer carbon matrix decorated with copper particles: enhancing the performance of Si as anode for Li-ion batteries. *ACS Nano.* **13,** 3054-3062 (2019).
54. Liu, Y. et al. Hierarchical engineering of porous P2-$Na_{2/3}Ni_{1/3}Mn_{2/3}O_2$ nanofibers assembled by nanoparticles enables superior sodium-ion storage cathodes. *Adv. Funct. Mater.* **30,** 1907837 (2020).
